# Eukaryotic translation factor eIF5A contributes to acetic acid tolerance in *Saccharomyces cerevisiae* via transcriptional factor Ume6p

**DOI:** 10.1186/s13068-021-01885-2

**Published:** 2021-02-08

**Authors:** Yanfei Cheng, Hui Zhu, Zhengda Du, Xuena Guo, Chenyao Zhou, Zhaoyue Wang, Xiuping He

**Affiliations:** 1 CAS Key Laboratory of Microbial Physiological and Metabolic Engineering, State Key Laboratory of Mycology, Institute of Microbiology, Chinese Academy of Sciences, Beijing, 100101 China; 2 College of Life Sciences, University of Chinese Academy of Sciences, Beijing, 100049 China

**Keywords:** *Saccharomyces cerevisiae*, Translation factor eIF5A, Transcription factor Ume6p, Acetic acid tolerance

## Abstract

**Background:**

*Saccharomyces cerevisiae* is well-known as an ideal model system for basic research and important industrial microorganism for biotechnological applications. Acetic acid is an important growth inhibitor that has deleterious effects on both the growth and fermentation performance of yeast cells. Comprehensive understanding of the mechanisms underlying *S. cerevisiae* adaptive response to acetic acid is always a focus and indispensable for development of robust industrial strains. eIF5A is a specific translation factor that is especially required for the formation of peptide bond between certain residues including proline regarded as poor substrates for slow peptide bond formation. Decrease of eIF5A activity resulted in temperature-sensitive phenotype of yeast, while up-regulation of eIF5A protected transgenic *Arabidopsis* against high temperature, oxidative or osmotic stress. However, the exact roles and functional mechanisms of eIF5A in stress response are as yet largely unknown.

**Results:**

In this research, we compared cell growth between the eIF5A overexpressing and the control *S. cerevisiae* strains under various stressed conditions. Improvement of acetic acid tolerance by enhanced eIF5A activity was observed all in spot assay, growth profiles and survival assay. eIF5A prompts the synthesis of Ume6p, a pleiotropic transcriptional factor containing polyproline motifs, mainly in a translational related way. As a consequence, *BEM4, BUD21* and *IME4*, the direct targets of Ume6p, were up-regulated in eIF5A overexpressing strain, especially under acetic acid stress. Overexpression of *UME6* results in similar profiles of cell growth and target genes transcription to eIF5A overexpression, confirming the role of Ume6p and its association between eIF5A and acetic acid tolerance.

**Conclusion:**

Translation factor eIF5A protects yeast cells against acetic acid challenge by the eIF5A-Ume6p-Bud21p/Ime4p/Bem4p axles, which provides new insights into the molecular mechanisms underlying the adaptive response and tolerance to acetic acid in *S. cerevisiae* and novel targets for construction of robust industrial strains.

## Background

eIF5A, originally discovered and identified as an eukaryotic translation initiation factor (eIF), is a small (16–18 kDa) cellular protein containing the unique amino acid hypusine [N^*ϵ*^-(4-amino-2-hydroxybutyl)lysine], which is formed from a specific lysine residue in eIF5A via posttranslational modification [1–6]. The hypusine modification of eIF5A involves two enzymatic reactions, in which deoxyhypusine synthase (DHS) catalyzes the transfer of a *n*-butylamine moiety from the polyamine spermidine to one specific lysine residue of the eIF5A precursor to form deoxyhypusine intermediate, then deoxyhypusine hydroxylase (DOHH) catalyzes the hydroxylation of deoxyhypusine to form hypusine-containing, biologically active eIF5A [4, 6]. Both eIF5A precursor and its hypusine modification are highly conserved and essential in all archaea and eukaryotes, suggesting the importance of active eIF5A in these organisms [5–13]. eIF5A was initially characterized to function in initiation of protein synthesis for its ability to stimulate the synthesis of methionyl-puromycin, a model reaction indicating the synthesis of the first peptide bond [1–3]. Such function was also observed for eIF5A in *Saccharomyces cerevisiae* [14]. However, several researches confirmed the role for eIF5A in translation elongation rather than translation initiation, as its depletion resulted in the increased polysomes in ribosome run-off experiments, yielding polysome profiles similar to those of elongation factor mutants [15–17], and functional interactions with structural components of the 80S ribosome and elongation factor eEF2 were observed for eIF5A [15, 18]. Only about 30% decrease in the overall protein synthesis was observed in eIF5A rapid-deletion yeast cells [14, 19], indicating that eIF5A appears to facilitate translation of some specific mRNAs rather than involvement in general protein synthesis. The specific requirement for eIF5A in translation of sequence-specific proteins that contain at least three consecutive proline residues was revealed by in vivo assays in yeast and in vitro reconstituted translation assays, in which eIF5A binds near the E and P sites of the 80S ribosome with its hypusine residue pointing to the peptidyl transferase center to prevent ribosome stalling on consecutive proline codons and promote the translation through polyproline [20]. Proline-rich proteins occur widely in eukaryotic organisms, and the number and frequency of polyproline motifs increase with biological complexity of organisms, which indicate their functional significance [21–23]. However, the precise relations between the activity of eIF5A and the specialized target proteins, and their physiological roles remain elusive and need to be further elucidated.

*S. cerevisiae* is an ideal model system for eukaryote, and moreover is an important organism for biotechnological applications. Yeast cells might suffer from various environmental stresses during the process of fermentation, such as high temperature, oxidative stress, osmotic stress and inhibitors, which have deleterious effects on both the cell growth and fermentation performance [24–26]. Acetic acid is not only one of the crucial inhibitors in lignocellulosic hydrolysates, an important non-feedstock substrate, but a byproduct of *S. cerevisiae* fermentation as well, and also an effective preservative [25, 27–31]. Acidification of intracellular environment and accumulation of acetate anion are the main causative factors for the cytotoxicity of acetic acid, which result in arrest of cell growth and metabolic activity [28, 32–34]. Comprehensive understanding of the mechanisms underlying yeast response to acetic acid is always a focus of research and indispensable for development of robust yeast strains for industrial applications.

In *S. cerevisiae*, eIF5A is encoded by two homologous genes, *HYP2* and *ANB1*. *HYP2* is essential and mainly expresses under the aerobic condition, whereas the nonessential *ANB1* expresses only under the anaerobic condition [11, 35]. The *S. cerevisiae* eIF5A precursor is modified at the ε-amino group of Lys51 to form hypusine, which is catalyzed successively by DHS and DOHH encoded by *DYS1* and *LIA1,* respectively [10, 12, 36]. Mutation at Lys51 of eIF5A leads to lethal effect on yeast cells, while some other mutations in eIF5A result in temperature-sensitive phenotype [17, 37, 38]. In our previous comparative transcriptome analyses, downregulation of *DYS1* and *LIA1* was observed under heat stress for temperature-sensitive yeast strain (unpublished data). According to the above information, we hypothesized that eIF5A might involve in yeast response to environmental stresses. In this study, the role and functional mechanism of eIF5A in *S. cerevisiae* response to acetic acid were investigated.

## Results

### Overexpression of eIF5A enhances acetic acid tolerance of yeast cells

**Fig. 1:**
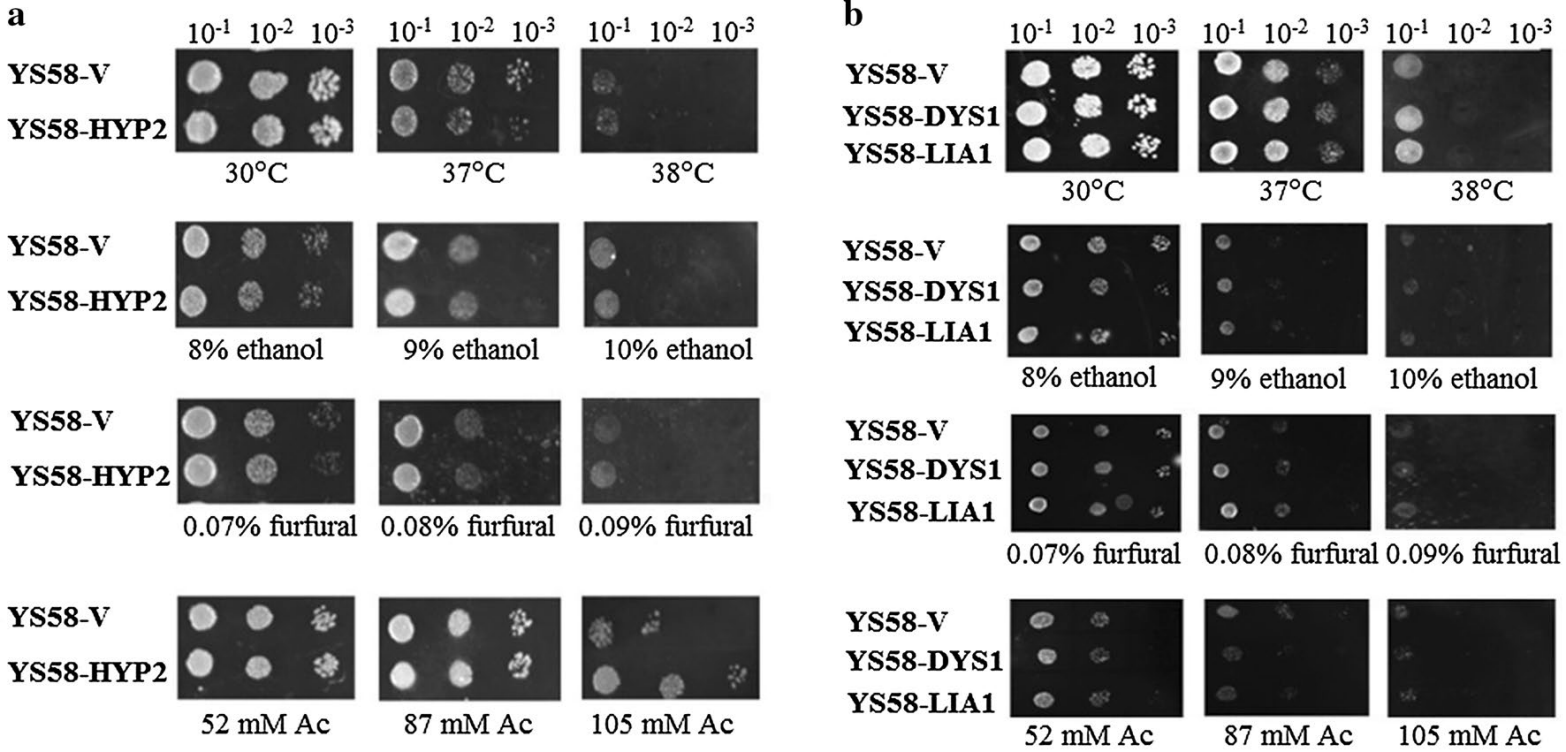
Comparison of cell growth among different yeast strains under various stressed conditions by spot dilution assay. **a** Comparison between *HYP2-*overexpressing strain and the control strain. **b** Comparison between yeast strains with enhanced hypusine modification and the control strain. Yeast cells were cultured on SC-Ura medium with or without inhibitors at 30 ℃ or other temperature

Cell growth of yeast strains overexpressing *HYP2*, *DYS1* or *LIA1* on synthetic complete medium SC-Ura under various stress conditions were compared with the control strain by spot assay first. No significant difference in growth was observed among these yeast strains under heat, furfural or ethanol stress (Fig. 1), while the eIF5A overexpressing strain YS58-HYP2 exhibited resistance to acetic acid compared with the control strain YS58-V (Fig. 1a).

**Fig. 2:**
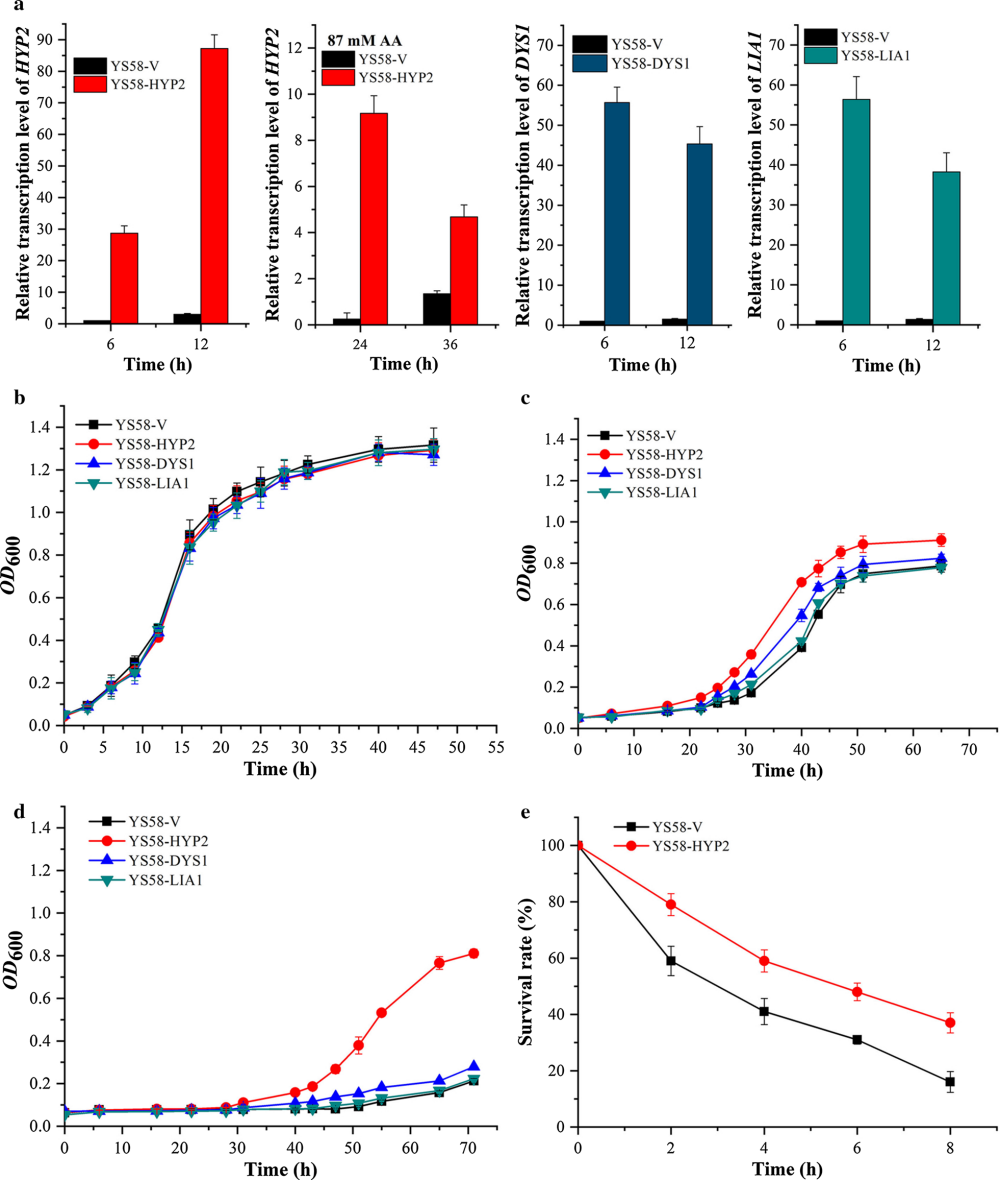
Expression levels of eIF5A relative genes and growth curves of different yeast strains. **a** Relative transcription levels of *HYP2*, *DYS1* and *LIA1* under conditions with or without acetic acid stress. The relative transcription levels of *HYP2*, *DYS1* and *LIA1* in the control strain YS58-V under non-stressed condition were defined as a value of 1. **b** Cell growth without acetic acid. **c** Cell growth in the presence of 87 mM acetic acid (pH 4.2). **d** Cell growth in the presence of 105 mM acetic acid (pH 4.2). **e** Survival assay of different yeast strains after 210 mM acetic acid treatment (pH 4.2). Data are presented as the means of the results of three independent experiments. Error bars represent standard deviations

To validate the results of spot assay, the transcription levels of related genes in various yeast strains and cell growth in liquid SD-Ura medium with different concentrations of acetic acid were determined (Fig. 2). The enhanced transcription of *HYP2*, *DYS1* or *LIA1* in strain YS58-HYP2, YS58-DYS1 or YS58-LIA1 was confirmed, respectively, by qRT-PCR (Fig. 2a). There was no obvious growth difference among these yeast strains under non-stressed condition (Fig. 2b). Cell growth of all strains was impaired under acetic acids stress, but less influence on eIF5A-overexpressing strain YS58-HYP2 was observed, which displayed much shorter lag time and higher growth efficiency than the control strain YS58-V in the presence of 87 mM acetic acid (Fig. 2c). When the concentration of acetic acid was increased to 105 mM, growth of the control strain YS58-V was suppressed almost completely, whereas cells of YS58-HYP2 could still grow with around 50% growth rate of that in the absence of acetic acid after a longer lag time of about 30 h (Fig. 2d). Enhancement of *DYS1* expression only improved the cell growth slightly under acetic acid stressed conditions, while overexpression of *LIA1* displayed similar growth profiles to the control strain under the various conditions (Fig. 2c and d). In the presence of 87 mM acetic acid, the transcription level of *HYP2* in YS58-HYP2 were 1.8 and 6.1 times of those of the control strain, respectively, in early-exponential growth phase and mid-exponential growth phase (Fig. 2a), indicating a correlation between acetic acid tolerance and higher transcription level of eIF5A. In further survival assay, higher survival rate was obtained for strain YS58-HYP2 in the presence of 210 mM acetic acid (Fig. 2e). In the preliminary fermentation, higher glucose utilization in YS58-HYP2 than in YS58-V was observed in the presence of 87 mM acetic acid (Additional file 1: Figure S1). These results suggest that eIF5A protects yeast cells against acetic acid damage in *S. cerevisiae*.

### The possible targets of eIF5A mediating acetic acid tolerance

**Fig. 3:**
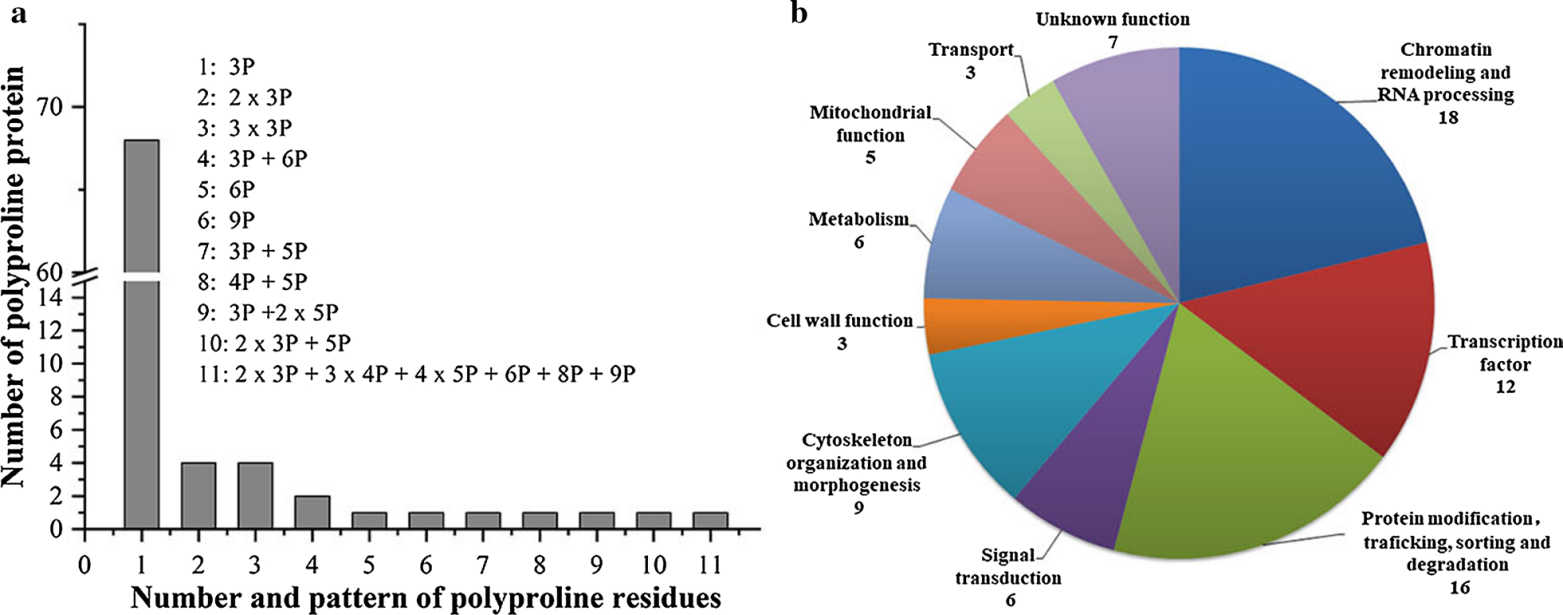
Patterns of polyproline residues (PPP) and the functional ontology diagram of *S. cerevisiae* polyproline proteins involved possibly in tolerance to acetic acid. **a** Numbers and patterns of polyproline residues. **b** Functional ontology

It has been reported that eIF5A is required for synthesis of proteins containing polyproline motifs through stimulating peptide bond formation between prolines in translation elongation step [20]. Thus, we proposed that enhancement of eIF5A might promote the translation of proline repeat-rich proteins which involve in response to acetic acid stress. To test this hypothesis, bioinformatics retrieval and analysis were conducted firstly. Five hundred and fifty eight yeast proteins with polyproline stretches (at least triple proline residues, PPP or 3P) were found in *Saccharomyces* Genome Database (SGD, http://www.yeastgenome.org/download-data/sequence). In the functional screening of EUROSCARF nonessential genes deletion collection, there were 216 genes whose deletion caused the sensitive phenotype to 0.5% acetic acid at pH 4.2 [39]. About 650 genes whose deletion caused the sensitive phenotype to 70, 90 and 110 mM acetic acid at pH 4.5 were identified in the screening of the same mutant collection [29]. In another screening of the same mutant collection for genes involved in the positive and negative regulation of acetic acid-induced programmed cell death (PCD) by analysis the amount of culturable cells in the presence of 400 mM acetic acid at pH 3.0, there were 409 genes whose deletion caused the sensitive phenotype to acetic acid [40]. After consistency analysis of the three datasets to exclude the repeats, a total of 1031 genes were obtained and regarded as the potential genes involved in tolerance to acetic acid. Among the 558 polyproline proteins coding genes and 1031 genes possibly related to acetic acid tolerance, 85 genes common to the two datasets were identified and clustered according to the Gene ontology (GO) annotations available on SGD based on specific biological process and molecular function of the genes, which are mainly involved in chromatin remodeling, transcriptional regulation, RNA processing, protein modification, intracellular trafficking and sorting or degradation, signal transduction, cytoskeleton organization and morphogenesis, cell wall function, metabolism and mitochondrial function (Fig. 3) (Additional file 1: Table S1). Among the 85 common genes, there are 17 genes encoding proteins with multiple PPP motifs (≥ 2 × 3P) or longer consecutive proline residues (≥ PPPP), which might require much higher eIF5A activity for peptide bond formation between consecutive proline residues. Hence, they were chosen for further investigation for their more dependence on eIF5A activity.

### Influence of eIF5A levels on the abundance of polyproline proteins related probably to acetic acid tolerance

**Fig. 4:**
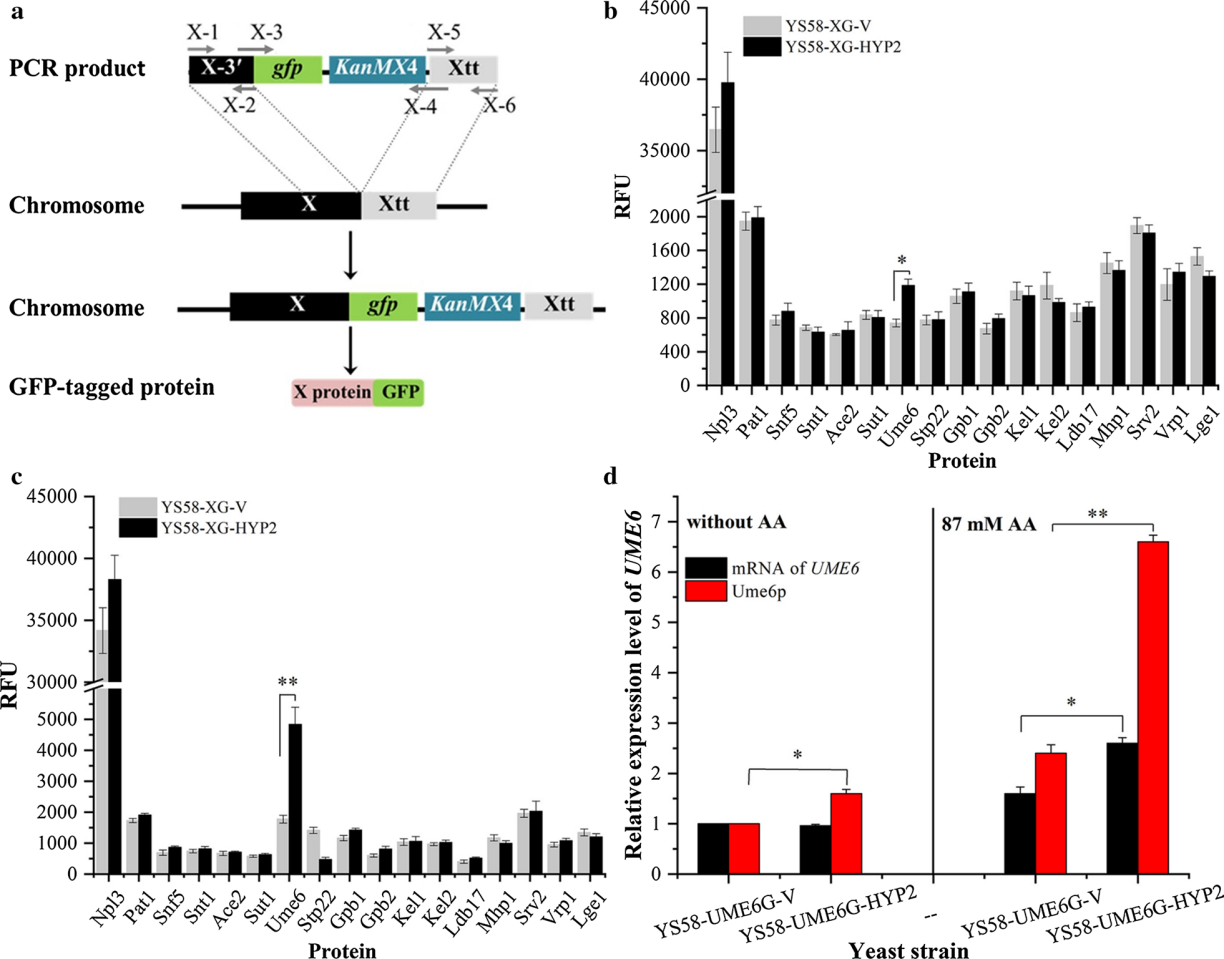
Quantification of the abundance of polyproline proteins involved possibly in tolerance to acetic acid. **a** GFP-tagged strategy for determination the abundance of polyproline proteins. Three pairs of primers were designed to amplify integrated DNA fragment to allow in-frame fusion of the GFP tag at the C-terminal coding region of the gene (X represents each gene name of interest). X-1/X-2 and X-5/X-6 were designed to amplify upstream and downstream homologous arms (about 400 bp) for recombination in chromosome, X-3/-X-4 were designed to amplify the GFP-marker cassette. X-2 and X-3, X-4 and X-5 shared complementary sequences, respectively. Then the gene-specific oligonucleotide fragment was obtained by overlap PCR of the three segments. **b** The relative abundance of polyproline proteins under condition without acetic acid. **c** The relative abundance of polyproline proteins in the presence of 87 mM acetic acid. **d** Comparison of mRNA level of *UME6* and protein level of Ume6p. Yeast cells were cultured under conditions with or without 87 mM acetic acid (pH 4.2) for 4 h. The relative transcription level of *UME6* or protein level of Ume6p in YS58-UME6G-V under non-stressed condition was defined as a value of 1, respectively. Data are presented as the means of the results of three independent experiments. Error bars represent standard deviations (*P < 0.05, **P < 0.01)

GFP-tagged strategy was exploited to compare abundance of the polyproline proteins in yeast cells (Fig. 4a). A series of recombinant yeast strains were constructed, in which the native locus of the polyproline protein encoding gene on chromosome was replaced by a *gfp*-fused form to produce GFP-tagged target protein, respectively. Plasmid pYEAH was introduced into the above recombinant strains to obtain strains YS58-XG-HYP2, while the corresponding references YS58-XG-V with empty vector pYEA were also obtained. The enhanced transcription of *HYP2* similar to YS58-HYP2 was confirmed by qRT-PCR. The RFU of GFP in constructed strains was assayed and compared under 87 mM acetic acid stressed or non-stressed condition for 4 h and 8 h, respectively, to characterize the levels of target proteins (Fig. 4b, c). The changing trend of abundance of each protein at the two time points was almost accordant. For ease of comparison, results of cultivation for 4 h in the absence or presence of 87 mM acetic acid were presented. Among them, the abundance of Ume6p increased largely in *HYP2*-overexpressing strain under both conditions, which were 1.6-fold and 2.7-fold under non-stressed condition and acetic acid stress for 4 h compared with the respective control strain (Fig. 4b, c). Moreover, induction of Ume6p by acetic acid was observed, which confirmed its roles in *S. cerevisiae* response to acetic acid.

To further validate the role of eIF5A in synthesis of Ume6p, both the transcription level of *UME6* and the protein level of Ume6p were analyzed (Fig. 4d). Under non-stressed condition, there was no obvious difference in mRNA level of *UME6*, but 67% increase in Ume6p abundance was observed in eIF5A overexpressing strain compared to the control strain, which indicated that the enhanced eIF5A activity supported 67% increase in translation efficiency of *UME6* mRNA. Increases in both mRNA level and Ume6p abundance occurred in strains YS58-UME6G-V and YS58-UME6G-HYP2 in the presence of 87 mM acetic acid, which were 1.6 and 2.4 times in the control strain, 2.7 and 4.1 times in eIF5A overexpressing strain of those under non-stressed condition. The translation efficiency of *UME6* mRNA was 69% higher in YS58-UME6G-HYP2 than YS58-UME6G-V in the presence of 87 mM acetic acid. These results confirm that the elevated eIF5A promotes the synthesis of Ume6p in mainly a translational related way.

### Transcriptional activation of key targets of Ume6p

**Fig. 5:**
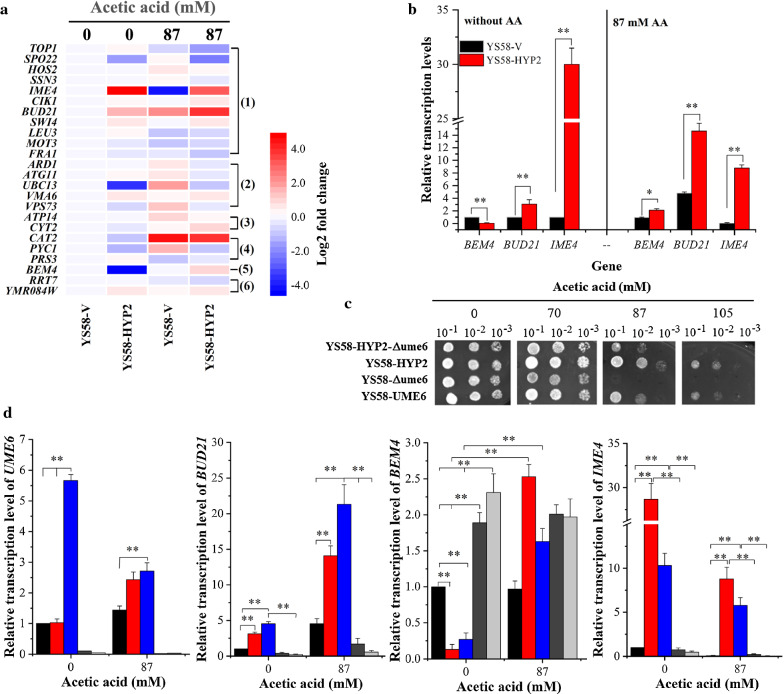
Ume6p mediates the effect of *HYP2* overexpression on yeast cells response to acetic acid. **a** Direct targets of Ume6p involved in acetic acid response. The log2 values of relative transcription levels were compared. (1) Genes involved in chromatin remodeling, transcription and RNA processing. (2) Genes involved in protein modification, trafficking and sorting. (3) Genes involved in mitochondrial function. (4) Genes involved in carbohydrate metabolism. (5) Gene involved in remodeling and maintaining cell wall architecture. (6) Unknown function. **b** Transcription levels of Ume6p direct targets. **c** Effect of Ume6p activity on cell growth upon acetic acid stress. **d** Effect of Ume6p activity on transcription of *BEM4*, *BUD21* and *IME4*. Yeast cells were cultured under conditions with or without 87 mM acetic acid (pH 4.2) for 4 h. The relative transcription of target gene in YS58-V under non-stressed condition was defined as a value of 1, respectively. Data are presented as the means of the results of three independent experiments. Error bars represent standard deviations (*P < 0.05, **P < 0.01)

Ume6p is regarded as a pleiotropic transcriptional factor involved in response to nutritional changes or stresses, meiosis and hyphal development [29, 41–44]. To further verify the role of Ume6p in acetic acid response, the target genes regulated potentially by Ume6p were predicted by using the YEASTRACT database (http://www.yeastract.com). A total of 1481 potential target genes were searched, in which 170 genes were regarded as the direct targets for the consensus sequence in promoters. One hundred and seventy-six Ume6p target genes whose deletion led to sensitive phenotype to acetic acid were found by consistency comparison between target genes of Ume6p and genes potentially related to acetic acid tolerance, among which 24 genes were the direct targets of Ume6p (Fig. 5a). Divergences in transcriptional profiles of the direct target genes of Ume6p were observed between the eIF5A overexpressing strain and the control strain under both conditions. The transcription of *BUD21*, *CAT2*, *PYC1*, *VPS73* and *UBC13* was enhanced largely, but the transcription of *IME4* was repressed severely by acetic acid in the control strain, while the mRNA levels of other genes displayed no obvious changes between non-stressed and stressed conditions. In eIF5A overexpressing yeast cells, genes *BUD21* and *IME4* were up-regulated, while transcription of *PYC1* and *SPO22* was repressed compared to the control cells under both non-stressed and stressed conditions. Transcription of *BEM4* and *UBC13* were repressed in eIF5A overexpressing yeast cells cultured under non-stressed condition, but they were de-repressed in the presence of acetic acid. The other genes showed no obvious change in mRNA levels across different strains and culture conditions. Under acetic acid stressed condition, eIF5A overexpressing strain produced much higher level of mRNA of *BEM4*, *BUD21* and *IME4* than the control strain (Fig. 5b), which suggested that Bem4p, Bud21p and Ime4p might mediate the acetic acid tolerance endowed by eIF5A overexpression. Among them, *BUD21* is the sole gene up-regulated by both acetic acid (4.8-fold) and eIF5A (3.1-fold), suggesting its importance in yeast response to acetic acid challenge.

Furthermore, influence of *UME6* deletion or overexpression on yeast cell growth and transcription of key target genes were investigated. Susceptible phenotype to acetic acid was observed in strains YS58-ume6 and YS58-HYP2-ume6, while protective effect of enhanced Ume6p on yeast cells against acetic acid stress occurred (Fig. 5c), suggesting the significance of Ume6p. However, strain YS58-ume6 was more sensitive to acetic acid than strain YS58-HYP2-ume6, while strain YS58-HYP2 was more resistant to acetic acid than strain YS58-UME6, indicating there may be other eIF5A-dependent proteins involving in protection yeast cells from acetic acid stress. Reduction of *BUD21* and *IME4* mRNA levels was observed in *ume6Δ* mutants YS58-ume6 and YS58-HYP2-ume6, while mRNA levels of *BUD21* and *IME4* were increased by overexpression of *UME6*, no matter acetic acid stress or not (Fig. 5d) (Additional file 1: Figure S2). Activation of *BEM4* occurred in *ume6Δ* mutants, while *BEM4* was repressed by enhanced expression of *UME6* under non-stressed condition and de-repressed under acetic acid stressed condition (Fig. 5d). Similar growth profiles and transcription patterns of Ume6p target genes in *HYP2* overexpression and *UME6* overexpression genetic backgrounds suggest that Ume6p plays key function in tolerance to acetic acid endowed by eIF5A.

## Discussion

As an important factor involved in translation, eIF5A is essential for eukaryotic vitality. However, it is necessary only for synthesis of some specific proteins containing polyproline motifs (at least three consecutive proline residues, PPP) [20], or other poor substrates for formation of peptide bond, such as glycine, arginine, lysine, aspartic acid or glutamic acid [45–47]. The proteins under eIF5A control relate to various biological processes, including cell wall integrity and cytoskeleton organization, cycle and cell differentiation, mRNA synthesis and turnover, and so on [23, 45, 48]. However, the key factors mediating the association between eIF5A activity and specific functional groups are poorly understood. Temperature-sensitive phenotype was observed in eIF5A and its hypusine modification mutants [17, 37, 38, 49]. Up-regulation of eIF5A in *Rosa chinensis* under high temperature, oxidative or osmotic stress condition, and its protective effect on transgenic *Arabidopsis* against above stresses were observed [50]. These results suggest that eIF5A might involve in response to environmental stresses. In this study, the effect of enhanced eIF5A activity on the tolerance of yeast cells to various stresses was investigated. Cell growth profiles under different stressed conditions indicate that the enhancement of eIF5A protects yeast cells from acetic acid damage rather than other stresses, which is a novel role for eIF5A involved in stress response.

Acetic acid is one of the common inhibitors that yeast cells suffer during growth and fermentation processes. Programmed cell death can be induced by lethal concentrations of acetic acid by apoptosis or necrosis, while sublethal concentrations of acetic acid may cause lower cell growth after extended period of growth arrest [34]. Hence, prolongation of lag phase is a typical characteristic of acetic acid damage, while short-term adaptation in robust strains facilitates the fermentation performance of *S. cerevisiae* in the presence of acetic acid [31, 51, 52]. Much shorter lag period and higher growth efficiency was observed for *HYP2*-overexpressing strain YS58-HYP2 than the control strain YS58-V in the presence of 87 mM acetic acid, and growth of the control strain YS58-V was suppressed almost completely whereas cells of YS58-HYP2 could still grow after an extended lag period of about 30 h when the concentration of acetic acid was increased to 105 mM (Fig. 3). The robustness of *HYP2*-overexpressing strain YS58-HYP2 under acetic acid stress confirmed the involvement of eIF5A in tolerance to acetic acid.

Proline residues are regarded as the poorest substrates for peptide bond formation for the unique geometric conformations, and translation of polyproline peptides might require much more eIF5A activity [20]. So, we proposed that the improvement of yeast tolerance to acetic acid by overexpression of *HYP2* might be mediated by some specific polyproline proteins. Shared analysis between polyproline proteins-encoding genes and candidate genes leading to sensitivity to acetic acid after deletion [29, 39, 40] was conducted to identify a total of 85 common genes, among which the abundance of protein encoded by *UME6* was increased significantly by overexpression of *HYP2*, suggesting that Ume6p might play a positive role in associating eIF5A activity and tolerant phenotype to acetic acid.

Ume6p (Unscheduled Meiotic Expression 6), initially identified as a repressor of meiosis-specific genes, is a global pleiotropic transcriptional factor, which has been found to play positive or negative roles in diverse biological processes, including carbon and nitrogen metabolism, DNA repair, meiosis and hyphal development, cell wall biogenesis and maintenance [41–44]. More than one thousand genes in *S. cerevisiae* genome are putative under the control of Ume6p, in which genes with upstream repressor site 1 (URS1) within promoters are regarded as the direct targets [42, 44, 53, 54]. The regulatory roles of Ume6p during meiosis have been well studied, in which Ume6p interacts with URS1 motif and recruits the conserved histone deacetylase Rpd3 through the co-repressor Sin3 and the chromatin-remodeling factor Isw2 to repress the target genes [41, 54]. In response to environmental changes, acetylation of Ume6p occurs, leading to its release from promoters and the subsequent degradation, allowing the induction of target gene transcription [54–56]. However, the transcription levels of some genes with URS1 motif did not change in *ume6Δ* mutant [54], while some genes without the URS1 were bound and positively regulated by Ume6p [57], suggesting that the roles and regulatory mechanisms of Ume6 are much more complex than those in meiosis. The involvement of Ume6p in acetic acid response was first reported by genome-wide screening *S. cerevisiae* haploid mutants sensitive to acetic acid [29], but the roles and the underlying mechanisms of this polyphonic transcription factor in acetic acid response are yet to be explored and confirmed. Among 24 of Ume6p direct target genes leading to sensitivity to acetic acid after deletion, transcription of *BEM4, BUD21* and *IME4* was up-regulated largely by eIF5A overexpression under acetic acid stress, indicating that Bem4p, Bud21p and Ime4p might involve in acetic acid tolerance caused by eIF5A overexpression. Further analyses of *UME6* deletion or overexpression confirmed the dependency of *BUD21*, *IME4* and *BEM4* transcription on Ume6p. These results suggest that eIF5A-Ume6p switch regulates *S. cerevisiae* tolerance to acetic acid probably by promoting ribosome biogenesis through Bud21p, a component of the small ribosomal subunit processosome [58], remodeling and maintaining cell wall architecture through Bem4p activation of the cell wall integrity pathway [59], and Ime4p-mediated epitranscriptional regulation by m^6^A methylation of target mRNAs [60–62]. In particular, *BUD21* is the sole gene up-regulated by both acetic acid (4.8-fold) and eIF5A (3.1-fold), suggesting its importance in protecting yeast cells from acetic acid challenge. The further investigation needs to be conducted to reveal the exact roles of Bem4p, Bud21p and Ime4p in *S. cerevisiae* response to acetic acid, especially in that associated with eIF5A.

## Conclusions

In conclusion, the involvement of translation factor eIF5A in *S. cerevisiae* response to acetic acid stress is confirmed in this study. eIF5A prompts the synthesis of a pleiotropic transcription factor Ume6p, which facilitates the transcription of multiple target genes to maintain normal biological processes, including ribosome biogenesis, cell wall biogenesis, epitranscriptional regulation and so on. Results in this study provide new insights into the molecular mechanisms underlying the adaptive response to acetic acid in *S. cerevisiae* by implicating eIF5A-Ume6p-Bud21p/Ime4p/Bem4p axles as signaling modules to endow yeast cells tolerance to acetic acid. Nevertheless, it is noteworthy that eIF5A-dependent proteins include more than those investigated in this study, the roles of other proteins under the control of eIF5A in acetic acid response need to be further investigated. On the other hand, only genes reported currently as both the direct target of Ume6p for the existence of the consensus sequence and involving in response to acetic acid were investigated in this study. Whether other target genes of Ume6p function in tolerance to acetic acid endowed by eIF5A requires further investigation. Moreover, in view of the significance of m^6^A methylation modification of mRNA in regulation of gene expression, the physiological association of m^6^A methylation with adaptive response and tolerance to acetic acid will be also explored in the future works.

## Materials and methods

### Strains and growth conditions

*Escherichia coli* DH5α (*supE44 ∆lacU169 (φ80lacZ∆M15) hsdR17 recA1 endA1 gyrA96 thi-1 relA1*) was used as a general host for plasmid propagation. *S. cerevisiae* YS58 (MATα *flo1 leu2-3,112 his4-519 trp1-719 ura3-52*) [63] was used as the host for overexpression or deletion of target genes and construction of yeast strains with GFP-tagged proteins. The derivative yeast strains are listed in Additional file 1: Table S2. *E. coli* cells were grown at 37 °C in Luria–Bertani (LB) medium [64]. When necessary, 100 μg/mL of ampicillin was used in LB medium. Yeast cells were grown generally at 30 °C in YPD medium [65]. To screen or analyze yeast transformants, YPD medium containing 500 μg/mL of G418, synthetic complete medium (SC) containing 10 g/L glucose, 6.7 g/L yeast nitrogen base without amino acids, 40 mg/L histidine, 40 mg/L tryptophan, 40 mg/L leucine and 30 mg/L uracil or SC medium without uracil (SC-Ura) were also used. For preliminary fermentation, yeast cells were cultivated at 30 °C and 60 rpm in synthetic complete fermentation medium (SCFM) containing 100 g/L glucose, 6.7 g/L yeast nitrogen base without amino acids, 5 g/L urea, 40 mg/L histidine, 40 mg/L tryptophan and 40 mg/L leucine.

### General DNA manipulations

General DNA manipulations in *E. coli* or *S. cerevisiae* were performed according to standard methods [64, 65]. Polymerase chain reaction (PCR) was conducted using high-fidelity DNA polymerase KOD plus according to the manufacturer’s instruction (TOYOBO, Japan). The oligonucleotide primers used in this study are listed in Additional file 1: Table S3. Purification of DNA fragments was performed using PCR Clean-up kit or DNA Gel Extraction kit (Axygen scientific Inc., USA). Total RNA was isolated from yeast cells by using the hot phenol method [65]. Gene transcription was analyzed by quantitative real-time PCR (qRT-PCR) using the Quant one-step qRT-PCR kit (SYBR Green) and LightCycler 96 System (Roche, Switzerland). Data were processed by the second-derivative maximum method of LightCycler 96 software SW1.1 with housekeeping gene *ACT1* as a control to calculate the relative transcription level of each target gene.

### Construction of recombinant yeast strains

The plasmids used in this study are listed in Additional file 1: Table S2. The regulatory element cassette containing the *ADH1* promoter, *ADH1* terminator and the multiple cloning sites (MCS) was amplified from plasmid pAUR123 (TaKaRa) by PCR with primer pair ADH1-F/ADH1-R. The amplified 990-bp DNA fragment was digested with *Hin*dIII/*Eco*RI, and then inserted into the corresponding sites of plasmid YEp352 [66] to generate plasmid pYEA1. For overexpression of *HYP2*, the 474 bp coding region of *HYP2* (Accession number NM_001178849.3) was amplified from *S. cerevisiae* YS58 genomic DNA with primer pair HYP2-F1/HYP2-R1. After digested by *Kpn*I/*Sac*I, *HYP2* was inserted into the corresponding sites of pYEA1 to generate plasmid pYEAH. Two DNA fragments P_ADH1_-1 and P_ADH1_-2 corresponding to the *ADH1* promoter region (Accession number KY704468) were amplified from genomic DNA of *S. cerevisiae* YS58 using primer pairs ADH1-DF/ADH1-DR and ADH1-DF/ADH1-LR, respectively, and then inserted into the *Eco*RI/*Bam*HI sites or *Eco*RI/*Kpn*I sites of plasmid YEp352 to generate plasmid pYEA2 and pYEA3. DNA fragment covering the coding region and terminator of *DYS1* (Accession number BK006934, 1449 bp) or *LIA1* (Accession number BK006943, 1327 bp) was amplified from genomic DNA of yeast YS58 by primer pair DYS1-F1/DYS-R1 or LIA1-F1/LIA1-R1, and inserted into plasmid pYEA2 or pYEA3 after digested by *Bam*HI/*Hind*III or *Kpn*I/*Hind*III, respectively, to generate recombinant plasmid pYEAD or pYEAL. All PCR products were verified by DNA sequencing, and all the plasmids were confirmed by restriction analysis.

Plasmids pYEAH, pYEAD and pYEAL were introduced into *S. cerevisiae* YS58 to generate recombinant strains YS58-HYP2, YS58-DYS1 and YS58-LIA1, respectively. As a control, *S. cerevisiae* YS58 was transformed with empty vector pYEA1 to generate control strain YS58-V. All transformants were screened on SC-Ura plates using *URA3* as the selectable marker and confirmed by PCR analysis. The genetic stability of yeast strains was analyzed as described previously [51].

To compare abundance of target proteins in yeast cells, GFP-tagged strategy was exploited. The 0.9 kb DNA fragment containing coding region of *gfp5* and *ADH1* terminator was amplified from plasmid pYCAGA using primers GFP-F and GFP-R, while 1.4 kb geneticin (G418) resistant gene *KanMX4* was amplified from plasmid pFA6a-KanMX4 [67] using primers KanMX-F and KanMX-R. The *gfp-KanMX* cassette was obtained by overlap PCR with primer pair GFP-F/KanMX-R. For expression of GFP-tagged proteins, three primer pairs (X-1/X-2, X-3/X-4 and X-5/X-6) were synthesized for each target gene. As indicated in Fig. 4a, primer pairs X-1/X-2, X-3/X-4 and X-5/X-6 were used to amplify the 3′-terminal coding sequence of target gene (X), the *gfp-KanMX* cassette and the terminator region of target gene (Xtt), respectively. Then a recombinant DNA fragment *X-gfp-KanMX-X*tt was obtained by overlap PCR with primer pair X1/X6. *S. cerevisiae* YS58 was transformed with each DNA fragment *X-gfp-KanMX-X*tt to generate a series of recombinant yeast strains, designated as YS58-XG, respectively, in which the native locus of target gene on chromosome was replaced by *gfp*-fused target gene by double cross-over recombination to produce GFP-tagged target protein.

For overexpression of *UME6*, DNA fragment covering 2511-bp coding region of *UME6* (Accession number NM_001180515.1) was amplified from genome of YS58 by PCR using primers UME6-ORF-F and UME6-ORF-R. After digested by *Sal*I/*Xba*I, the coding region of *UME6* was inserted into the corresponding sites of pYEA1 to generate plasmid pYEAU6, which then was transformed into *S. cerevisiae* YS58 to generate recombinant strain YS58-UME6. For deletion of *UME6*, a DNA fragment covering *URA3* flanked, respectively, by 5′-sequence and 3′-sequence of *UME6* (Accession number NC_001136.10) was obtained from plasmid pYEA1 by PCR using primers UME6-URA3-F and UME6-URA3-R, and then was transformed into *S. cerevisiae* YS58 to construct *UME6* knockout strain YS58-ume6 using *URA3* as the selective marker. Moreover, a DNA fragment covering *LEU2* flanked, respectively, by 5′-sequence and 3′-sequence of *UME6* was obtained from genome of YS58 by PCR using primers UME6-LEU2-F and UME6-LEU2-R, and then was used to generate *UME6* knockout strain YS58-HYP2-ume6 using *LEU2* as the selective marker.

### Stress tolerance assay

Tolerance of yeast cells to different stresses were compared by both spot dilution assay on solid media and growth profiles in liquid cultures [68]. Yeast cells were precultured in 2 mL of SC-Ura at 30 ºC for 18 h (the mid-exponential phase), and then harvested by centrifugation at 5000×*g* for 5 min, washed twice with sterile water and resuspended in sterile water to a final cell concentration equivalent to 0.1 of *OD*_600_. After diluted serially, 5 μL of each tenfold dilution (10^–1^–10^–3^) was spotted onto SC-Ura agar plates and agar plates containing different concentrations of acetic acid, ethanol or furfural, and then incubated at 30 °C for 48 h. For analysis of acetic acid tolerance, the pH of media was adjusted to 4.2 with 2 M HCl. For analysis of thermotolerance, yeast cells were incubated at different temperatures.

Acetic acid tolerance was also analyzed by liquid growth assay. Yeast cells precultured in SC-Ura medium at 30ºC for 18 h were inoculated into 50 mL SC-Ura medium containing 87 mM or 105 mM acetic acid, respectively, to a final cell density equivalent to about 0.05 of absorbance at 600 nm (*OD*_600_). Cultivation was performed at 30ºC with an agitation of 200 rpm, and the cell growth was monitored periodically. Three fundamental growth variables, growth lag (the intercept of the initial density and the slope), growth rate (the slope of the exponential phase of the growth curve) and growth efficiency (the total change in density for cells reached stationary phase) [49], were used to characterize the cell growth of different yeast strains under acetic acid stress.

For cell viability assay, yeast cells grown to the mid-exponential phase were harvested and resuspended in 5 mL of 210 mM acetic acid solution with adjusted pH of 4.2, in which the final cell concentration was controlled to 0.8 of *OD*_600_. As controls, same yeast cells were also resuspended in 5 mL of sterile water. Cell suspension was incubated at 30 °C with an agitation of 200 rpm. 100 μL of sample was drawn at different time and diluted serially. The serial dilutions were properly spread onto YPD plates. After 48 h of incubation at 30 °C, colony-forming units (CFU) were counted. Survival rate of yeast cells was calculated as the percentage of CFU at the specific time point and the CFU at starting time.

### Measurement of fluorescence intensity of GFP in yeast cells

For determination of the GFP fluorescence intensity, yeast cells were precultured in SC-Ura for 18 h at 30ºC with shaking, and then resuspended in 5 mL fresh SC-Ura supplied with or without 87 mM acetic acid (pH 4.2) to a final cell density equivalent to an *OD*_600_ of 0.8. Cultivation was performed at 30ºC with an agitation of 200 rpm. Yeast cells were harvested after 4 h and 8 h, respectively, washed twice with sterile water and resuspended in the same volume of sterile water. The fluorescence intensity (FI) of yeast cells was detected using a Synergy H4 hybrid reader (BioTek, USA) with excitation at 488 nm and emission at 509 nm. Meantime, *OD*_600_ was measured. Relative fluorescence unit (RFU), the rate of FI and *OD*_600_, was applied to characterize the abundance of GFP-tagged proteins. Translation efficiency was defined as the ratio of protein level to the corresponding mRNA level of a particular gene.

### Statistical analysis

All experiments were performed in three independent biological replicates, and at least three independent experiments were done on separate occasions. All data were analyzed statistically using Data Analysis and Graphing Software (OriginPro 7.5, OriginLab Corporation). Data are presented as the means of the results of three independent experiments and the standard deviations.

## Supplementary Information

**Additional file 1: Table S1.** Functional categories of polyproline proteins possibly involved in acetic acid response in *S. cerevisiae.* **Table S2.** Strains and plasmids used in this study. **Table S3.** Primers used in this study. **Figure S1.** Cell growth and glucose utilization of different yeast strains. Yeast cells precultured in SC-Ura medium at 30ºC with an agitation of 200 rpm for 20 h were inoculated into 100 mL synthetic complete fermentation medium (SCFM) containing 100 g/L glucose, 6.7 g/L yeast nitrogen base without amino acids, 5 g/L urea, 40 mg/L histidine, 40 mg/L tryptophan and 40 mg/L leucine to a final cell density equivalent to about 2.0 of absorbance at 600 nm (*OD*_600_). Cultivation was performed in the absence (YS58-V and YS-58-HYP2) or presence of 87 mM acetic acid (pH 4.2) (YS58-V (AA) and YS-58-HYP2 (AA)) at 30 °C with an agitation of 60 rpm. Samples were withdrawn periodically for analyses of cell growth and residual glucose. The level of residual glucose was detected using the dinitrosalicylic acid method. Data are presented as the means of the results of three independent experiments. Error bars represent standard deviations. **Figure S2.** Effect of Ume6p activity and eIF5A activity on transcription of *BEM4*, *BUD21* and *IME4*. Yeast cells were cultured under conditions with or without 87 mM acetic acid (pH 4.2) for 4 h. Total RNA was isolated from yeast cells by using the hot phenol method. Gene transcription was analyzed by quantitative real-time PCR (qRT-PCR) using the Quant one-step qRT-PCR kit (SYBR Green) and LightCycler 96 System (Roche, Switzerland). Data were processed by the second-derivative maximum method of LightCycler 96 software SW1.1 with housekeeping gene *ACT1* as a control to calculate the relative transcription level of each target gene. The relative transcription of target gene in YS58-V under non-stressed condition was defined as a value of 1, respectively. Data are presented as the means of the results of three independent experiments. Error bars represent standard deviations.

## Data Availability

The datasets supporting the conclusions of this article are included within the article and its additional file.

## Abbreviations

DHS: Deoxyhypusine synthase
DOHH: Deoxyhypusine hydroxylase
LB medium: Luria–Bertani medium containing (per liter) 5 g yeast extract, 10 g tryptone and 10 g NaCl
YPD medium: Yeast extract peptone dextrose medium containing (per liter)10 g yeast extract, 20 g peptone and 20 g glucose
SC medium: Synthetic complete medium containing (per liter) 6.7 g yeast nitrogen base without amino acids, 10 g glucose, 40 mg histidine, 40 mg tryptophan, 40 mg leucine and 30 mg uracil
SC-Ura: SC medium without uracil
AA: Acetic acid
PCR: Polymerase chain reaction
qRT-PCR: Quantitative real-time PCR
MCS: Multiple cloning sites
GFP: Green fluorescent protein
OD_600_: Optical density at 600 nm
CFU: Colony-forming units
FI: Fluorescence intensity
RFU: Relative fluorescence unit, the rate of FI and *OD*_600_
SGD: Saccharomyces Genome Database
GO: Gene ontology
URS1: Upstream repressor site 1
