# Supplementary material for: Eukaryotic translation factor eIF5A contributes to acetic acid tolerance in Saccharomyces cerevisiae via transcriptional factor Ume6p
Source: Biotechnol Biofuels. 2021 Feb 8;14:38. doi: 10.1186/s13068-021-01885-2 (PMC7869214; doi:10.1186/s13068-021-01885-2)
Supplement: Supplementary file 1 — Additional file 1: Table S1. Functional categories of polyproline proteins possibly involved in acetic acid response in S. cerevisiae. Table S2. Strains and plasmids used in this study. Table S3. Primers used in this study. Figure S1. Cell growth and glucose utilization of different yeast strains. Yeast cells precultured in SC-Ura medium at 30ºC with an agitation of 200 rpm for 20 h were inoculated into 100 mL synthetic complete fermentation medium (SCFM) containing 100 g/L glucose, 6.7 g/L yeast nitrogen base without amino acids, 5 g/L urea, 40 mg/L histidine, 40 mg/L tryptophan and 40 mg/L leucine to a final cell density equivalent to about 2.0 of absorbance at 600 nm (OD600). Cultivation was performed in the absence (YS58-V and YS-58-HYP2) or presence of 87 mM acetic acid (pH 4.2) (YS58-V (AA) and YS-58-HYP2 (AA)) at 30 °C with an agitation of 60 rpm. Samples were withdrawn periodically for analyses of cell growth and residual glucose. The level of residual glucose was detected using the dinitrosalicylic acid method. Data are presented as the means of the results of three independent experiments. Error bars represent standard deviations. Figure S2. Effect of Ume6p activity and eIF5A activity on transcription of BEM4, BUD21 and IME4. Yeast cells were cultured under conditions with or without 87 mM acetic acid (pH 4.2) for 4 h. Total RNA was isolated from yeast cells by using the hot phenol method. Gene transcription was analyzed by quantitative real-time PCR (qRT-PCR) using the Quant one-step qRT-PCR kit (SYBR Green) and LightCycler 96 System (Roche, Switzerland). Data were processed by the second-derivative maximum method of LightCycler 96 software SW1.1 with housekeeping gene ACT1 as a control to calculate the relative transcription level of each target gene. The relative transcription of target gene in YS58-V under non-stressed condition was defined as a value of 1, respectively. Data are presented as the means of the results of three independent experiments. E [file 13068_2021_1885_MOESM1_ESM.docx]

**Additional file 1**

**Table S1. Functional categories of polyproline proteins possibly involved in acetic acid response in *S. cerevisiae***

| Functional category | Gene | Polyproline motif | Function description |
| --- | --- | --- | --- |
| **Chromatin remodeling, transcription and RNA processing** | ARP8 | 3P | Component of chromatin-remodeling enzyme complexes |
|  | **SNF5** | **3P+2x5P** | Subunit of the SWI/SNF chromatin remodeling complex |
|  | NGG1 | 3P | Subunit of chromatin modifying histone acetyltransferase complexes |
|  | SIN3 | 3P | Component of both the Rpd3S and Rpd3L histone deacetylase complexes, involved in transcriptional repression or activation and maintenance of chromosomal integrity |
|  | **SNT1** | **2x3P** | Subunit of the Set3C deacetylase complex |
|  | AIR2 | 3P | Subunit of the TRAMP nuclear RNA surveillance complex, involved in nuclear RNA processing and degradation |
|  | BUR2 | 3P | Cyclin for the Sgv1p (Bur1p) protein kinase, involved in transcriptional regulation through phosphorylation of the largest subunit of RNA polymerase II (Rpo21p) and recruitment of Spt6p |
|  | CCR4 | 3P | Component of the CCR4-NOT transcriptional complex and the major cytoplasmic deadenylase, involved in regulation of gene expression and mRNA poly(A) tail shortening |
|  | CTK1 | 3P | Catalytic (alpha) subunit of C-terminal domain kinase I, involved in transcription, pre-mRNA 3' end processing and translational fidelity by phosphorylation of RNA pol II subunit Rpo21p and ribosomal protein Rps2p |
|  | DHH1 | 3P | Cytoplasmic DExD/H-box helicase, involved in mRNA decapping |
|  | HPR1 | 3P | Subunit of THO/TREX complexes and RNA Pol II complex, couple transcription elongation with mitotic recombination and with mRNA metabolism and export |
|  | MED2 | 3P | Subunit of the RNA polymerase II mediator complex |
|  | POP2 | 3P | RNase of the DEDD superfamily; subunit of the Ccr4-Not complex that mediates 3' to 5' mRNA deadenylation |
|  | **NPL3** | **2x3P+5P** | RNA-binding protein, involved in elongation, termination, transportation of poly(A) mRNA from nucleus to cytoplasm and pre-mRNA splicing |
|  | **PAT1** | **3P+6P** | Deadenylation-dependent mRNA-decapping factor |
|  | SPT20 | 3P | Subunit of the SAGA transcriptional regulatory complex |
|  | TOP1 | 3P | Topoisomerase I, involved in replication, transcription, and recombination |
|  | DEG1 | 3P | tRNA:pseudouridine synthase |
| **Transcription factor** | **ACE2** | **3x3P** | Transcription factor required for septum destruction after cytokinesis |
|  | AZF1 | 3P | Zinc-finger transcription factor |
|  | BAS1 | 3P | Myb-related transcription factor |
|  | CAT8 | 3P | Zinc cluster transcriptional activator |
|  | CST6 | 3P | Basic leucine zipper (bZIP) transcription factor |
|  | CYC8 | 3P | General transcriptional co-repressor or part of a transcriptional co-activator complex |
|  | INO2 | 3P | Transcription factor as the component of the heteromeric Ino2p/Ino4p basic helix-loop-helix transcription activator |
|  | MIG1 | 3P | Transcription factor involved in glucose repression |
|  | **SUT1** | **2x3P** | Transcription factor of the Zn(II)2Cys6 family |
|  | SWI1 | 3P | Transcriptional activator as the DNA binding component of the SBF complex (Swi4p-Swi6p) |
|  | TUP1 | 3P | General transcription repressor, involved in repressive chromatin structure formation |
|  | **UME6** | **3P+5P** | Pleiotropic transcriptional factor and component of the Rpd3L histone deacetylase complex |
| **Protein modification, traficking, sorting and degradation** | FPK1 | 3P | Ser/Thr protein kinase |
|  | HRK1 | 3P | Protein kinase |
|  | YPK1 | 3P | Serine/threonine protein kinase |
|  | RAV1 | 3P | Subunit of RAVE complex involved in assembly of the V-ATPase holoenzyme and transport between the early and late endosome and localization of TGN membrane proteins |
|  | SWA2 | 3P | Auxilin-like protein involved in vesicular transport and uncoating of clathrin-coated vesicles |
|  | VPS1 | 3P | Dynamin-like GTPase required for vacuolar sorting and late Golgi-retention of some proteins |
|  | VPS5 | 3P | Nexin-1 homolog required for localizing membrane proteins from a prevacuolar/late endosomal compartment back to late Golgi |
|  | VPS54 | 3P | Component of the GARP (Golgi-associated retrograde protein) complex required for the recycling of proteins from endosomes to the late Golgi |
|  | VPS64 | 3P | Protein required for cytoplasm to vacuole targeting of proteins |
|  | ATG11 | 3P | Adapter protein for pexophagy and the Cvt targeting pathway |
|  | ATG14 | 3P | Autophagy-specific subunit of phosphatidylinositol 3-kinase complex I required for localizing additional ATG proteins to the PAS and overflow degradation of misfolded proteins |
|  | RCY1 | 3P | F-box protein involved in recycling endocytosed proteins |
|  | UBP14 | 3P | Ubiquitin-specific protease |
|  | UBP3 | 3P | Ubiquitin-specific protease |
|  | **STP22** | **6P** | Component of the ESCRT-I complex involved in ubiquitin-dependent sorting of proteins into the endosome; |
|  | YME1 | 3P | Catalytic subunit of the i-AAA protease complex responsible for degradation of unfolded or misfolded mitochondrial gene products |
| **Signal transduction** | **GPB1** | **2x3P** | Multistep regulator of cAMP-PKA signaling |
|  | **GPB2** | **2x3P** | Multistep regulator of cAMP-PKA signaling |
|  | IRA2 | 3P | GTPase-activating protein involved in negative regulation of RAS by converting it from GTP- to GDP-bound form |
|  | PBS2 | 3P | MAP kinase kinase of the HOG signaling pathway |
|  | SCH9 | 3P | AGC family protein kinase phosphorylated by Tor1p and required for TORC1-mediated regulation |
|  | SSD1 | 3P | Translational repressor with a role in polar growth and wall integrity interacts with TOR pathway components |
| **Cytoskeleton organization, morphogenesis and cell wall function** | BEM2 | 3P | Rho GTPase activating protein involved in the control of cytoskeleton organization and cellular morphogenesis |
|  | KAR3 | 3P | Minus-end-directed microtubule motor |
|  | **KEL1** | **3x3P** | Protein required for proper cell fusion and cell morphology |
|  | **KEL2** | **3x3P** | Protein functions in a complex with Kel1p |
|  | **VRP1** | **2x3P+3x4P+4x5P**  **+6P+8P+9P** | Proline-rich actin-associated protein; involved in cytoskeletal organization and cytokinesis |
|  | **SRV2** | **3P+6P** | CAP (cyclase-associated protein) facilitating regulation of actin dynamics and cell morphogenesis |
|  | **MHP1** | **3x3P** | Microtubule-associated protein involved in microtubule organization |
|  | RRD1 | 3P | Peptidyl-prolyl cis/trans-isomerase involved in microtubule dynamics |
|  | TOS2 | 3P | Protein involved in localization of Cdc24p to the site of bud growth |
|  | **LDB17** | **9P** | Protein transiently recruited to actin cortical patches in a SLA1-dependent manner |
|  | CHS1 | 3P | Chitin synthase I |
|  | SHC1 | 3P | Sporulation-specific activator of chitin synthase III |
| **Mitochondrial function** | ATP5 | 3P | Subunit 5 of the stator stalk of mitochondrial F1F0 ATP synthase |
|  | MEF2 | 3P | Mitochondrial elongation factor involved in translational elongation |
|  | MSS51 | 3P | Specific translational activator for the mitochondrial COX1 mRNA |
|  | RRF1 | 3P | Mitochondrial ribosome recycling factor |
|  | SDH4 | 3P | Membrane anchor subunit of succinate dehydrogenase (SDH) involved in coupling the oxidation of succinate to the transfer of electrons to ubiquinone |
| **Metabolism** | PDA1 | 3P | E1 alpha subunit of the pyruvate dehydrogenase (PDH) complex |
|  | PFK2 | 3P | Beta subunit of heterooctameric phosphofructokinase |
|  | GPM2 | 3P | Homolog of Gpm1p phosphoglycerate mutase |
|  | THI20 | 3P | Trifunctional enzyme with hydroxymethylpyrimidine (HMP) kinase, HMP-phosphate (HMP-P) kinase and thiaminase activities |
|  | COQ8 | 3P | Protein required for ubiquinone biosynthesis |
|  | FAB1 | 3P | 1-phosphatidylinositol-3-phosphate 5-kinase |
| **Transport** | NHA1 | 3P | Na^+^/H^+^ antiporter |
|  | SPF1 | 3P | P-type ATPase, ion transporter of the ER membrane |
|  | LEM3 | 3P | Membrane protein of the plasma membrane and ER involved in translocation of phospholipids and alkylphosphocholine drugs across the plasma membrane |
| **Unknown function** | ERD1 | 3P | Predicted membrane protein required for lumenal ER protein retention |
|  | **LGE1** | **4P+5P** | Protein of unknown function |
|  | RBS1 | 3P | Protein of unknown function |
|  | SYH1 | 3P | Protein of unknown function that influences nuclear pore distribution |
|  | PBI1 | 3P | Putative protein of unknown function |
|  | PNS1 | 3P | Protein of unknown function |
|  | YMR196W | 3P | Putative protein of unknown function |

**Table S2. Strains and plasmids used in this study**

| **Strains/Plasmids** | **Genotype or description*** | **Reference/Source** |
| --- | --- | --- |
| **Strains** |  |  |
| *E. coli* DH5α | *sup*E44 ∆*lac*U169 (φ80*lac*Z∆M15) *hsd*R17 *rec*A1 *end*A1 *gyr*A96 *thi*-1 *rel*A1 | TAKARA |
| *S. cerevisiae* YS58 | *MATα flo1 leu2-3,112 his4-519 trp1-719 ura3-52* | [63] |
| YS58-V | YS58 (pYEA) | This study |
| YS58-HYP2  YS58-DYS1  YS58-LIA1  YS58-UME6  YS58-ume6  YS58-HYP2-ume6 | YS58 (pYEAH)  YS58 (pYEAD)  YS58 (pYEAL)  YS58 (pYEAU6)  YS58 derivative with *ume6Δ::URA3*  YS58-HYP2 derivative with *ume6Δ::LEU2* | This study  This study  This study  This study  This study  This study |
| YS58-STP22G-V/HYP2  YS58-UME6G-V/HYP2  YS58-NPL3G-V/HYP2  YS58-PAT1G-V/HYP2  YS58-SNF5G-V/HYP2  YS58-SNT1G-V/HYP2  YS58-ACE2G-V/HYP2  YS58-SUT1G-V/HYP2  YS58-GPB1G-V/HYP2  YS58-GPB2G-V/HYP2  YS58-KEL1G-V/HYP2  YS58-KEL2G-V/HYP2  YS58-LDB17G-V/HYP2  YS58-MHP1G-V/HYP2  YS58-SRV1G-V/HYP2  YS58-VRP1G-V/HYP2  YS58-LGE1G-V/HYP2 | YS58(pYEA1 or pYEAH, *gfp*-fused *STP22*)  YS58(pYEA1 or pYEAH, *gfp*-fused *UME6*)  YS58(pYEA1 or pYEAH, *gfp*-fused *NPL3*)  YS58(pYEA1 or pYEAH, *gfp*-fused *PAT1*)  YS58(pYEA1 or pYEAH, *gfp*-fused *SNF5*)  YS58(pYEA1 or pYEAH, *gfp*-fused *SNT1*)  YS58(pYEA1 or pYEAH, *gfp*-fused *ACE2*)  YS58(pYEA1 or pYEAH, *gfp*-fused *SUT1*)  YS58(pYEA1 or pYEAH, *gfp*-fused *GPB1*)  YS58(pYEA1 or pYEAH, *gfp*-fused *GPB2*)  YS58(pYEA1 or pYEAH, *gfp*-fused *KEL1*)  YS58(pYEA1 or pYEAH, *gfp*-fused *KEL2*)  YS58(pYEA1 or pYEAH, *gfp*-fused *LDB17*)  YS58(pYEA1 or pYEAH, *gfp*-fused *MHP1*) YS58(pYEA1 or pYEAH, *gfp*-fused *SRV1*)  YS58(pYEA1 or pYEAH, *gfp*-fused *VRP1*)  YS58(pYEA1 or pYEAH, *gfp*-fused *LGE1*) | This study  This study  This study  This study  This study  This study  This study  This study  This study  This study  This study  This study  This study  This study  This study  This study  This study |
| **Plasmids** |  |  |
| YEp352 | *E. coli-S. cerevisiae* shuttle vector, Amp^r^ for *E. coli* and *URA3* for *S. cerevisiae* | [66] |
| pAUR123 | *E. coli-S. cerevisiae* shuttle vector, Amp^r^ for *E. coli* and ABA resistance for *S. cerevisiae*, carrying *ADH1* promoter and terminator | TAKARA |
| pFA6a-kanMX4 | *E. coli*-Yeast shuttle vector, Amp^r^ for *E. coli* and G418 resistance for *S. cerevisiae* | [67] |
| pYEA1  pYEA2  pYEA3 | YEp352 derivative with insertion of *ADH1* promoter and terminator between *Eco*RI and *Hind*III  YEp352 derivative with insertion of *ADH1* promoter and terminator between *Eco*RI and *Bam*HI  YEp352 derivative with insertion of *ADH1* promoter and terminator between *Eco*RI and *Kpn*I | This study  This study  This study |
| pYEAH  pYEAD  pYEAL  pYEAU6  pYCAGA | pYEA1 derivative with insertion of *HYP2* coding region between *Kpn*I and *Sac*I  pYEA2 derivative with insertion of *DYS1* coding region between *Bam*HI and *Hind*III  pYEA3 derivative with insertion of *LIA1* coding region between *Kpn*I and *Hind*III  pYEA1 derivative with insertion of *UME6* coding region between *Sal*I and *Xba*I  Plasmid with coding region of GFP and *ADH1* terminator | This study  This study  This study  This study  Our lab |

*Amp^r^, ampicillin resistance

**Table S3. Primers used in this study**

| **Primer** | **Sequence (5′-3′)^a^** | **Purpose** | |
| --- | --- | --- | --- |
| ADH1-F | GGCCAAGCTTctccctaacatgtagg | PCR of *ADH1* promoter and terminator |  |
| ADH1-R | GCCGGAATTCgtgtggaagaacgattac |  |  |
| HYP2-F1 | CGGGGTACCatgtctgacgaagaacatac | PCR of *HYP2* coding region for expression  PCR of *ADH1* promoter  PCR of *ADH1* promoter  PCR of *ADH1* promoter  PCR of *DYS1* coding region for expression  PCR of *LIA1* coding region for expression  PCR of *gfp5* and *ADH1* terminator  PCR of *gfp5* and *ADH1* terminator  PCR of *KanMX*  PCR of *KanMX*  Overlap PCR of *gfp*-fused *NPL3*  Overlap PCR of *gfp*-fused *PAT1*  Overlap PCR of *gfp*-fused *SNF5*  Overlap PCR of *gfp*-fused *SNT1*  Overlap PCR of *gfp*-fused *ACE2*  Overlap PCR of *gfp*-fused *SUT1*  Overlap PCR of *gfp*-fused *UME6* |  |
| HYP2-R1  ADH1-DF  ADH1-DR  ADH1-LR  DYS1-F1  DYS1-R1  LIA1-F1  LIA1-R1  GFP-F  GFP-R  KanMX-F  KanMX-R  NPL3-1  NPL3-2  NPL3-3  NPL3-4  NPL3-5  NPL3-6  PAT1-1  PAT1-2  PAT1-3  PAT1-4  PAT1-5  PAT1-6  SNF5-1  SNF5-2  SNF5-3  SNF5-4  SNF5-5  SNF5-6  SNT1-1  SNT1-2  SNT1-3  SNT1-4  SNT1-5  SNT1-6  ACE2-1  ACE2-2  ACE2-3  ACE2-4  ACE2-5  ACE2-6  SUT1-1  SUT1-2  SUT1-3  SUT1-4  SUT1-5  SUT1-6  UME6-1  UME6-2 | GCCGAGCTCttaatcggttctagcagctt  CCGGAATTCATAACCGCTAGAGTACTTTGA  CGCGGATCCTGTATATGAGATAGTTGATTG  CGGGGTACCTGTATATGAGATAGTTGATTG  CGCGGATCCatgtccgatatcaacgaaaa  CCCAAGCTTaataggaatacaaaagccgct  CGGGGTACCatgtctactaactttgaaaa  CCCAAGCTTaggtctaaggcctcattgatt  GGTGGTGGTGGTTCTATGAGTAAAGGAGAAGAACT  GGCAAGCTAAACAGATCTGGGCGAATTTCTTATGATTTATG  CATAAATCATAAGAAATTCGCCCAGATC TGTTTAGCTTGCC  GCGGCGTTAGTATCGAATCGACAGC  TGACAATCCTCCACCAATCAG  CCTGGTTGGTGATCTTTCACGTGG  CCACGTGAAAGATCACCAACCAGGGGTGGTGGTGGTTCTATGAG  TACTGGAGTGAAGTCGGGACTCGCGCGGCGTTAGTATCGAATCG  GCGAGTCCCGACTTCACTCCAGTAG  AGGGGAAACTGGTCTACAGC  TTGGAAAGTAAGATTCAGCTG  TTAGTTCTGATATTTCACCATCGCG  CGCGATGGTGAAATATCAGAACTAAAG GGTGGTGGTGGTTCTATGAG  TCTTGCGTTACTATGGTGGTATTGCGGCGTTAGTATCGAATCG  AATACCACCATAGTAACGCAAGAG  GTCTTGCTTGTGCCTGGTAC  GCAGGCCAGATAGACCTAAG  TTCTGTGTGTTATTGTTACTGC  GCAGTAACAATAACACACAGAATACGGTGGTGGTGGTTCTATGAG  ACTCCGCATCGTATAACACGAGCGGCGTTAGTATCGAATCG  TCGTGTTATACGATGCGGAGTC  TTCCTATATGTCTGGCTGCTTG  GAATGCAATTGACATTGGCC  TTATTTTCCTTTTTAGATAAAAACTGCTC  TTATCTAAAAAGGAAAATAATAATGGTGGTGGTTCTATGAG  GCTCGAGAGTTTCACCAACCATGCGGCGTTAGTATCGAATCG  ATGGTTGGTGAAACTCTCGAGC  GAGTAACGAGTGAATTCATG  TGCGACTTTCCCGGTTGCAC  GCATCAGTTTCGTTTGAAAGGGTG  CACCCTTTCAAACGAAACTGATGCTCTCGGTGGTGGTGGTTCTATGAG  GCCCTTAAGACTACAGTGTACGGCGGCGTTAGTATCGAATCG  CGTACACTGTAGTCTTAAGGGCC  CCCTCGCTATAAGATCAACC  AAGTTGTGACAAATGTCGTT  AATCAATGCTTTTATAGTCATC  GATGACTATAAAAGCATTGATTTTGGTGGTGGTGGTTCTATGAG  CTCTTATTATGTAGGGTTATACATGGCGGCGTTAGTATCGAATCG  CATGTATAACCCTACATAATAAGAGTATAC  TTGAATAGAGATGAGCACTCG  GCGAATCCTCAACTTCGTCAGCT  GCTCTTCTTTTGGCCTCTTTTG |  |  |
| UME6-3  UME6-4  UME6-5  UME6-6  GPB1-1  GPB1-2  GPB1-3  GPB1-4  GPB1-5  GPB1-6  GPB2-1  GPB2-2  GPB2-3  GPB2-4  GPB2-5  GPB2-6  KEL1-1 | GAGGCCAAAAGAAGAGCAATGAAAAAAAAAGGTGGTGGTGGTTCTATGAG  GAAATTACATGGTAGTACATACGCGGCGTTAGTATCGAATCG  GTATGTACTACCATGTAATTTCAAAGAC  CAATGAATTTTGCAAATCCAACGTG  ACACAAGAGGAATCTGATGG  GGCTAGATTCATGCTTGGTAGAATG  TCATTCTACCAAGCATGAATCTAGCCAACGGTGGTGGTGGTTCTATGAG  GGAAACGCTACTATTGTTGTATAGTGCGGCGGCGTTAGTATCGAATC  CGCACTATACAACAATAGTAGCGTTTCC  CGTTCTGCCATGGTCTATCTAC  TTGGATAGTGTGAACGTAGGTC  TGCACTAGGATTTACGCTAGG  TTTCCTAGCGTAAATCCTAGTGCAGGTGGTGGTGGTTCTATGAG  CTGGTATTGCGAAGGAGATTATGCGGCGTTAGTATCGAATC  ATAATCTCCTTCGCAATACCAGCG  ATGGGCAGTCCTTGTATTCGAC  ATTAAGTCAAGATGTATTAG | Overlap PCR of *gfp*-fused *GPB1*  Overlap PCR of *gfp*-fused *GPB2*  Overlap PCR of *gfp*-fused |  |
| KEL1-2  KEL1-3  KEL1-4  KEL1-5  KEL1-6  KEL2-1  KEL2-2  KEL2-3  KEL2-4  KEL2-5  KEL2-6  STP22-1  STP22-2  STP22-3  STP22-4  STP22-5  STP22-6  LDB17-1  LDB17-2  LDB17-3  LDB17-4  LDB17-5  LDB17-6  MHP1-1  MHP1-2  MHP1-3  MHP1-4  MHP1-5  MHP1-6  SRV2-1  SRV2-2  SRV2-3  SRV2-4  SRV2-5  SRV2-6 | TAGTAGATCGCTGTCAGCATCTTC  TGAAGATGCTGACAGCGATCTACTAGGTGGTGGTGGTTCTATGAG  TTTGTCACGTTACTATTTCTCTCCTGCGGCGTTAGTATCGAATC  AGGAGAGAAATAGTAACGTGACAAATAG  ACGAACAGCTTCAACGTACC  CTAATTTACTCGACAATAAC  TTTGTTCGAGAGTATATAGTTT  AAACTATATACTCTCGAACAAAAAAAA GGTGGTGGTGGTTCTATGAG  GGCTTAGTTGGCAATTATATTC GCGGCGTTAGTATCGAATC  GAATATAATTGCCAACTAAGCC  ATTATTCAAGCCAACATGGTG  GACTATGTAGCGGACAAGATACTGACG  CCCGATAACGGTGAGGTGATTCG  CGAATCACCTCACCGTTATCGGGTGGTGGTGGTTCTATGAGT  CTGGGTTAGTGAGCTCAGATGTTCTGCGGCGTTAGTATCGAATCGACAGC  AGAACATCTGAGCTCACTAACCCAG  ACCTCGGTGTTGCACACGACCAAC  GGGTTCTGATTCCGCTATTAAAG TGGAGTTCCACATTTTCTTGATGGAGG  CCTCCATCAAGAAAATGTGGAACTCCAAAAGGTGGTGGTGGTTCTATGAGT  TGTCATTAGATACTGCAGTGAGAGTAGGCGGCGTTAGTATCGAATCGACAGC  CTACTCACTGCAGTATCTAATGACAC  TCCGTTTCTGACCCATTAACAG  GTCGGTGGTGGCGATTTGCCAGTG  TACTACTGTTCCGCGCATCCTGG  TCCAGGATGCGCGGAACAGTAGTACTAAAGGTGGTGGTGGTTCTATGAG  GTAAATGTCTGAGGGCTCCTTATTATATGCGGCGTTAGTATCGAATCGAC  ATATAATAAGGAGCCCTCAGACATTTAC  TTGTTGAGGTGTATTGAAGTTCAC  AGATGAGTCTATCTTCATAGGTAAATG  ACCAGCATGTTCGAAAACAGCAGATT  AATCTGCTGTTTTCGAACATGCTGGTGGTGGTGGTGGTTCTATGAG  GATCAAGTAGTCACTTAGACGCTCCTCGGCGGCGTTAGTATCGAATCGAC  CGAGGAGCGACTAAGTGACTACTTGATC  AGGTGATGAACCAAAAGCACG | *KEL1*  Overlap PCR of *gfp*-fused  *KEL2*  Overlap PCR of *gfp*-fused  *STP22*  Overlap PCR of *gfp*-fused  *STP22*  Overlap PCR of *gfp*-fused  *MHP1*  Overlap PCR of *gfp*-fused  *SRV2* |  |
| VRP1-1  VRP1-2  VRP1-3  VRP1-4  VRP1-5  VRP1-6  LGE1-1  LGE1-2  LGE1-3  LGE1-4  LGE1-5  LGE1-6  UME6-ORF-F  UME6-ORF-R  UME6-URA3-F  UME6-URA3-R  UME6-LEU2-F  UME6-LEU2-R  ACT1-F  ACT1-R | GTGTCTACATCAATACCATCAC  CGTAAATAATGTTAAGTCCAATGGC  GCCATTGGACTTAACATTATTTACGGGTGGTGGTGGTTCTATGAG  GTTCAGTAGTCTTGTAAGCTCATCGCGCGGCGTTAGTATCGAATCGAC  GCGATGAGCTTACAAGACTACTGAAC  CTGATTCATCTGAAGAAATCGG  GCTTATCCATATTCCAGCAG  CTGCATTAATAACAATGAGTCCAGC  AGCTGGACTCATTGTTATTAATGCAGGGTGGTGGTGGTTCTATGAG  CCTAATCGGGCTTAACCTAGCAGTACCGCGGCGTTAGTATCGAATCGAC  GGTACTGCTAGGTTAAGCCCGATTAGG  CAAGGAAGTTTAGAAAGGCG ATGTCGACatgctagacaaggcgcgctc  CCGTCTAGAttgtacacgagaattgacgt  GCGCACAGGAACTAGGACACTACCGCACTCAAACCATTTGCATGGACCTTAACTCACGATcatctgtgcggtatttcacac  TCATAGTGACAGTCCAATTTCAACCTTTCACAGTTGAAACAGTGCGGTCTTTCCTCGGTACACTTCcaatgatgggtaacaagagc  GCGCACAGGAACTAGGACACTACCGCACTCAAACCATTTGCATGGACCTTAACTCACGATatcttgaccgcagttaactg  TCATAGTGACAGTCCAATTTCAACCTTTCACAGTTGAAACAGTGCGGTCTTTCCTCGGTACACTTCaatggtcaggtcattgagtg  CGCTGCTCAATCTTCTTC  GGCTCTGAATCTTTCGTTAC | Overlap PCR of *gfp*-fused  *VRP1*  Overlap PCR of *gfp*-fused  *LGE1*  PCR of *UME6* coding region for expression  PCR of *URA3* flanked by *UME6* homologous arms  PCR of *LEU2* flanked by *UME6* homologous arms  qRT-PCR of *ACT1* |  |
| HYP2-F2 | AGTTCCAGTTGTCAAGAGA | qRT-PCR of *HYP2* |  |
| HYP2-R2 | CTTAGTGTCACCGTCCAT |  |  |
| DYS1-F2  DYS1-R2  LIA1-F2  LIA1-R2  UME6-F  UME6-R  BEM4-F | TTCGTTAAGGTTCAAGGTAT  CAATAATCTCACAGGCAGTA  GTCGTCAGAGAATCTTGTA  ATTAGCAGTTGGAGCATA  CGCCAACAATCATCACTA  GCTCCATCACTAACTTCAT  CCTGTATTGCGATGGATA | qRT-PCR of *DYS1*  qRT-PCR of *LIA1*  qRT-PCR of *UME6*  qRT-PCR of *BEM4* |  |
| BEM4-R | CTTAGTTGTTCTGCTTGAC |  |  |
| BUD21-F  BUD21-R  TOP1-F  TOP1-R  UBC13-F  UBC13-R  CIK1-F  CIK1-R  SPO22-F  SPO22-R  HOS2-F  HOS2-R  SSN3-F  SSN3-R  FRA1-F  FRA1-R  IME4-F  IME4-R  LEU3-F  LEU3-R  MOT3-F  MOT3-R  SWI4-F  SWI4-R  ARD1-F  ARD1-R  ATG11-F  ATG11-R  UMA6-F  VMA6-R  ATP14-F  ATP14-R  CYT2-F  CYT2-R  VPS73-F  VPS73-R  CAT2-F  CAT2-R  PRS3-F  PRS3-R  PYC1-F  PYC1-R  RRT7-F  RRT7-R  YMR084W-F  YMR084W-R | CTATAAGGCTGGAACAATC  ACCTCTGTCTCATTCACT  TTCAAGACGAAGCGGAACC  GAGGCGATGGTAATGACGAC  CCAATGGAAGCACCGAAAG  TTGAATGGACAGCAGGACG  AAAGACCTACAAGACACCCA  TGAGAGGAACAACGGCAA  TTTCGGACAAACACCAACC  TTTCTCTGAATGCTGCTGTG  CATCCAATGAAGCCGTTTCG  CCTCTGGGCAGTTTGTTTG  GAATACGAACTACGAGGGAAG  TTCTCTACAGGCACTCTGAG  GTGCTGGAAATGGGTCTGT  ATGTTCGTCACAACTGGGC  TGGGTAGACTGCTTGGAGT  GGTCGTGTATTTGGCTCTTTC  TCAGATAAGGACGAACCCG  GTATTCCACGAAGTGTCGG  ATACAAACAACGCTCCTCAG  GATGGTTAGGTGCTGTCGTA  AGTTCAAGGTGGGTATGGTAG  TTGCTTCTCTTTGGTGGAGG  CAACCTTCATAACCTACCCG  ACTTGATTGTCCTGCCGT  GAAGTTGCCGTTATCAGGAG  AAGACGACATAGACCCGAC  ATAAGAGACCAATCCAGTGG  GGCAAAGTATCAAACCAACC  TCTGCCATTGAGACTGTG  TTTGTGGTGGGTTCCAAG  ACCCGTCGGAGAAACAAT  GTTCCAAACCCGCTCATT  AGTGGGTATTTCGTGTGGC  GGGCTATTCCTTGTGTCAAC  ACTTACCATCATTACCCGTG  GCATACTCTTTCAATCGGTC  GGAGAATCTGTTAGGGACC  GTAATAGGAGCACGAGACTT  ACACCAGGTAGATTTCATCC  ACCCACGGAGTCAATAACT  AAAGGATTATGCGGGAGAAG  GCGGTTGAGAGTAAATGATG  GACAAGAGGAGAAATCATCG  CGAAATAAAGGGCAGGTTC | qRT-PCR of *BUD21*  qRT-PCR of *TOP1*  qRT-PCR of *UBC13*  qRT-PCR of *CIK1*  qRT-PCR of *SPO22*  qRT-PCR of *HOS2*  qRT-PCR of *SSN3*  qRT-PCR of *FRA1*  qRT-PCR of *IME4*  qRT-PCR of *LEU3*  qRT-PCR of *MOT3*  qRT-PCR of *SWI4*  qRT-PCR of *ARD1*  qRT-PCR of *ATG11*  qRT-PCR of *VMA6*  qRT-PCR of *ATP14*  qRT-PCR of *CYT2*  qRT-PCR of *VPS73*  qRT-PCR of *CAT2*  qRT-PCR of *PRS3*  qRT-PCR of *PYC1*  qRT-PCR of *RRT7*  qRT-PCR of *YMR084W* |  |

^a^Restriction sites or complementary sequences are underlined.


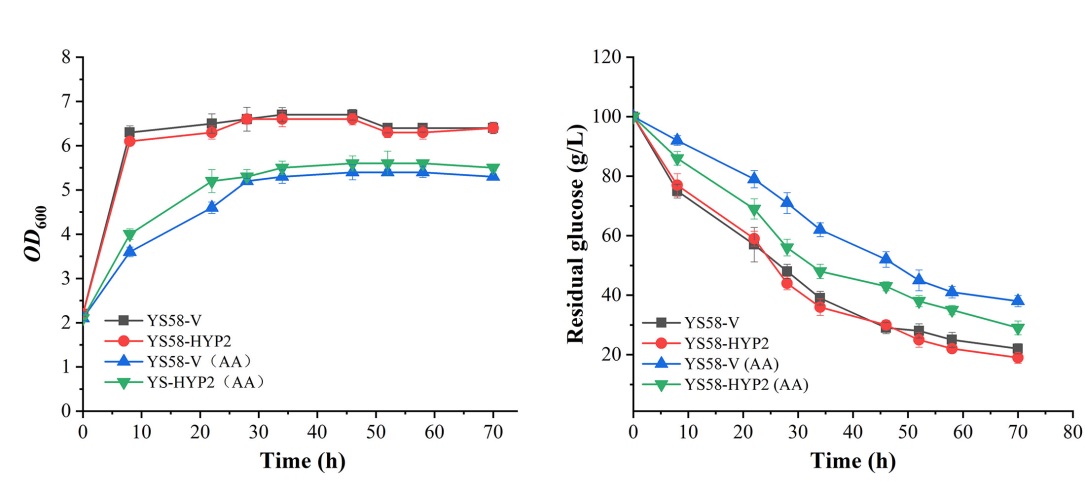


Figure S1 Cell growth and glucose utilization of different yeast strains. Yeast cells precultured in SC-Ura medium at 30ºC with an agitation of 200 rpm for 20 h were inoculated into 100 mL synthetic complete fermentation medium (SCFM) containing 100 g/L glucose, 6.7 g/L yeast nitrogen base without amino acids, 5 g/L urea, 40 mg/L histidine, 40 mg/L tryptophan and 40 mg/L leucine to a final cell density equivalent to about 2.0 of absorbance at 600 nm (*OD*_600_). Cultivation was performed in the absence (YS58-V and YS-58-HYP2) or presence of 87 mM acetic acid (pH 4.2) (YS58-V (AA) and YS-58-HYP2 AA)) at 30°C with an agitation of 60 rpm. Samples were withdrawn periodically for analyses of cell growth and residual glucose. The level of residual glucose was detected using the dinitrosalicylic acid method. Data are presented as the means of the results of three independent experiments. Error bars represent standard deviations.


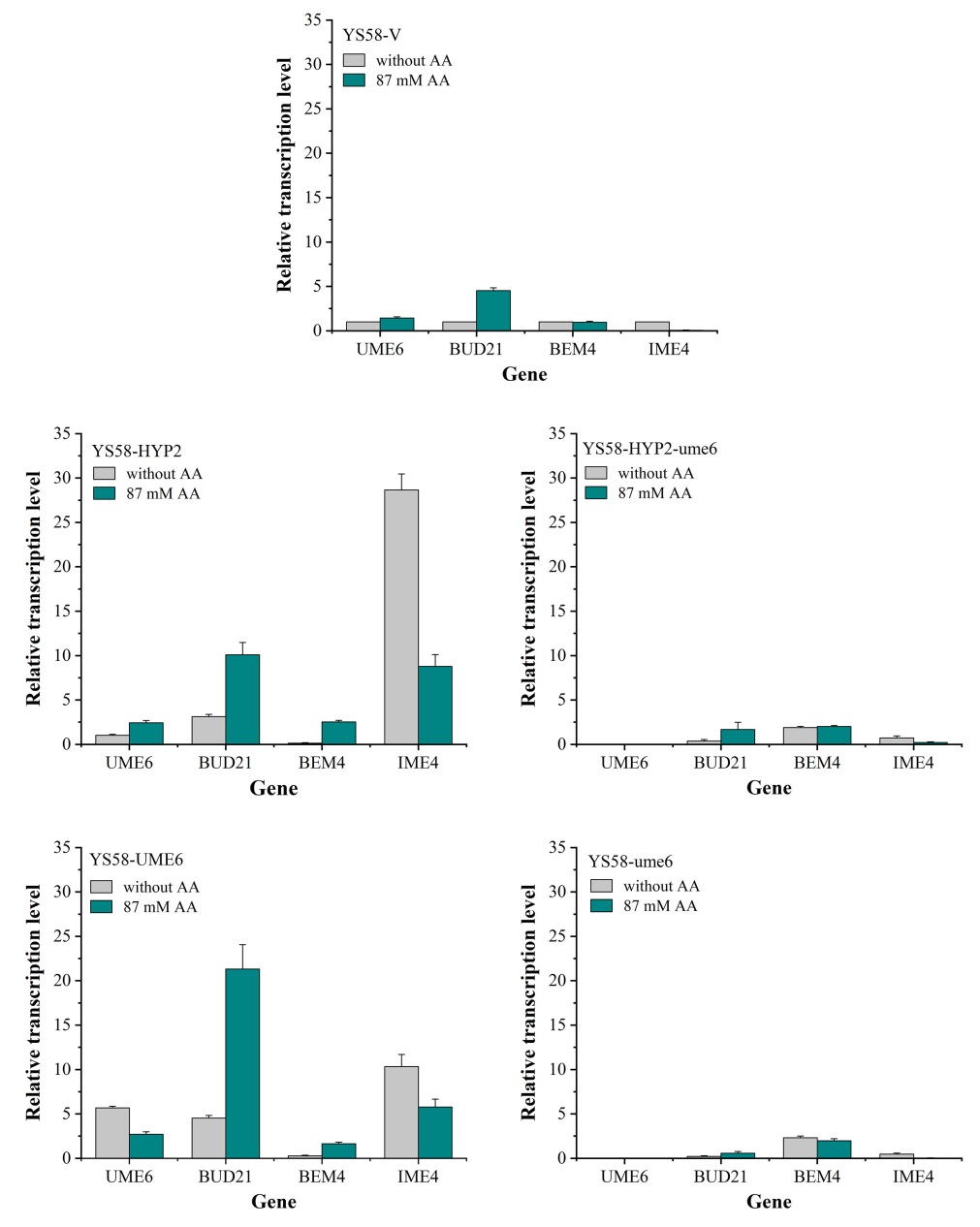


Figure S2 Effect of Ume6p activity and eIF5A activity on transcription of *BEM4*, *BUD21* and *IME4*. Yeast cells were cultured under conditions with or without 87 mM acetic acid (pH 4.2) for 4 h. Total RNA was isolated from yeast cells by using the hot phenol method. Gene transcription was analyzed by quantitative real-time PCR (qRT-PCR) using the Quant one-step qRT-PCR kit (SYBR Green) and LightCycler 96 System (Roche, Switzerland). Data were processed by the second-derivative maximum method of LightCycler 96 software SW1.1 with housekeeping gene *ACT1* as a control to calculate the relative transcription level of each target gene. The relative transcription of target gene in YS58-V under non-stressed condition was defined as a value of 1 respectively. Data are presented as the means of the results of three independent experiments. Error bars represent standard deviations.
